# Endoscopic papillectomy combined with endoscopic retrograde cholangio-pancreatography for duodenal gangliocytic paraganglioma: A case report

**DOI:** 10.1097/MD.0000000000036662

**Published:** 2023-12-15

**Authors:** Wenpin Cai, Weitao Hu, Taiyong Fang

**Affiliations:** a Department of Gastroenterology, The Second Affiliated Hospital of Fujian Medical University, Quanzhou, Fujian, P.R. China.

**Keywords:** duodenal gangliocytic paraganglioma (DGP), endoscopic papillectomy (EP), endoscopic retrograde cholangiopancreatography (ERCP)

## Abstract

**Rationale:**

Gangliocytic paraganglioma is a rare tumor that can occur in several organs throughout the body. Gangliocytic paraganglioma of the main duodenal papilla is even rarer. This study analyzes and discusses the endoscopic management of a case of gangliocytic paraganglioma of the main duodenal papilla and reviews the relevant literature. It is hoped that this study will increase clinicians’ awareness of this disease.

**Patient concerns:**

Electron endoscopy reveals a duodenal main papillary tumor, and the patient desires further clarification of the nature of the tumor and the next step in the treatment plan.

**Diagnoses:**

Duodenal gangliocytic paraganglioma.

**Interventions:**

As the patient lesion was located in the main duodenal papilla, we successfully performed endoscopic minimally invasive treatment of the tumor by endoscopic papillectomy combined with endoscopic retrograde cholangiopancreatography.

**Outcomes:**

The patient was discharged after the postoperative removal of the nasobiliary drain and returned to the hospital 2 months later to have the biliary stent removed; the patient was in good general condition at follow-up.

**Lessons:**

For duodenal main papillary tumor, we need to be alert to the possibility of gangliocytic paraganglioma. Since the tumor is located in the submucosa of the juxta-abdominal region, the preoperative biopsy positivity rate is low, and the tumor is often adjacent to or involves the biliopancreatic duct, endoscopic resection combined with endoscopic retrograde cholangiopancreatography can be considered for diagnosis and treatment.

## 1. Introduction

Duodenal gangliocytic paragangliomas (DGP) are rare tumors that often occur near the duodenal papilla. These tumors are considered benign and lymph node metastasis (LNM) is a rare occurrence, even more so with distant metastatic disease. Resection of the tumor is the only definitive treatment.^[1]^ This study analyzes and discusses the endoscopic management of a case of gangliocytic paraganglioma of the main duodenal papilla and reviews the relevant literature. This study was approved by the Clinical Research Ethics Committee of the Second Affiliated Hospital of Fujian Medical University ([2021]221). The patient has given consent for the case to be published.

## 2. Case report

**Figure 1.**
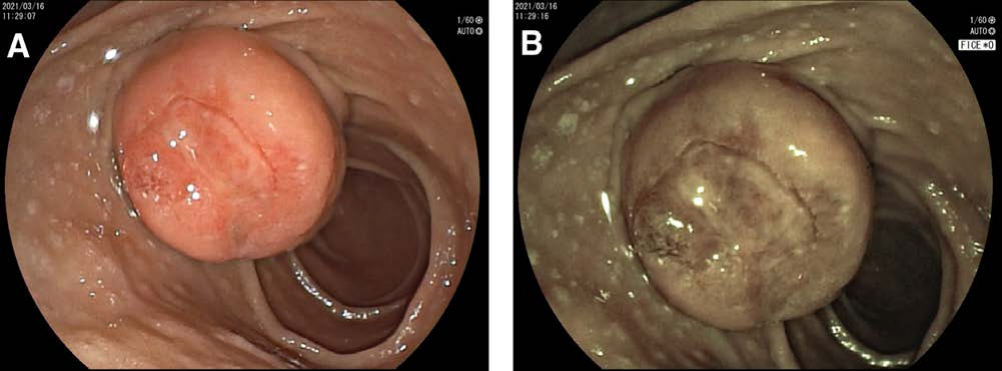
Rounded elevated mass in the main papilla of the descending duodenum. (A) Endoscopic white light chart. (B) Diagram in endoscopic FICE mode. FICE = Flexible Spectral Imaging Color Enhancement.

**Figure 2.**
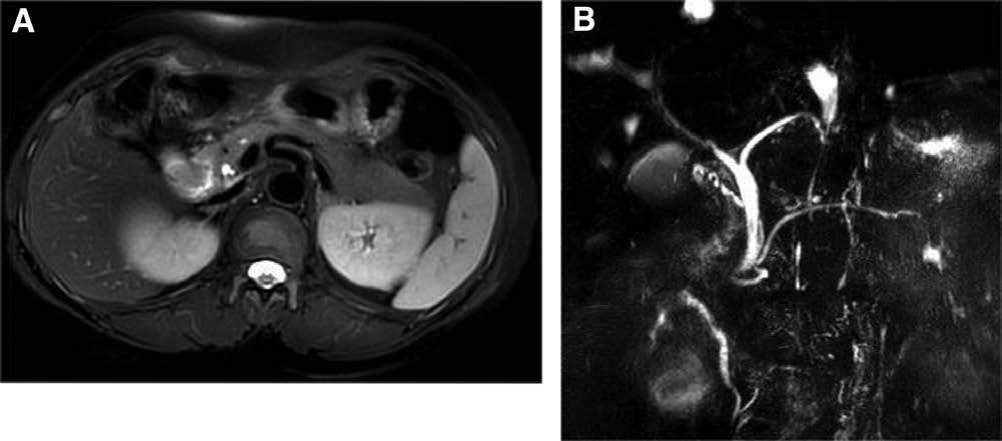
MRI and MRCP of the upper abdomen. (A) MRI enhancement. (B)MRCP. MRCP = magnetic resonance cholangiopancreatography, MRI = magnetic resonance imaging.

**Figure 3.**
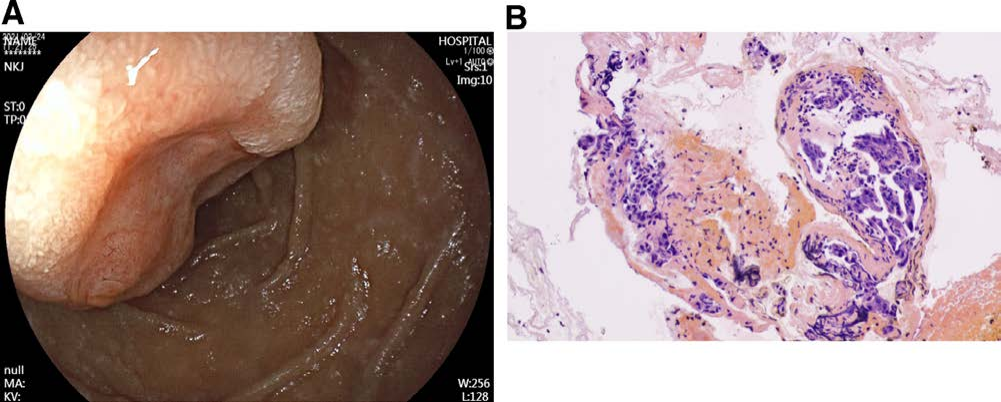
Pathological biopsy. (A) Endoscopic white light chart. (B) Pathologic examination after biopsy.

**Figure 4.**
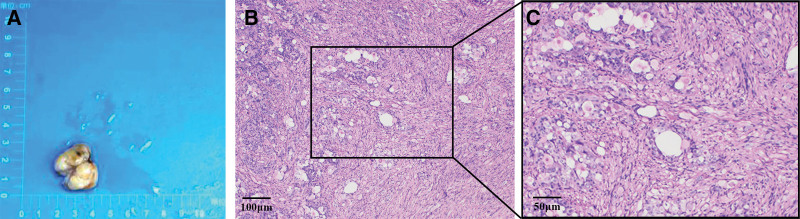
Postoperative pathology after EP combined with ERCP. (A) Bulk specimen (B) HE staining −10X (C) HE staining −20X. EP = endoscopic papillectomy, ERCP = endoscopic retrograde cholangiopancreatography.

**Figure 5.**
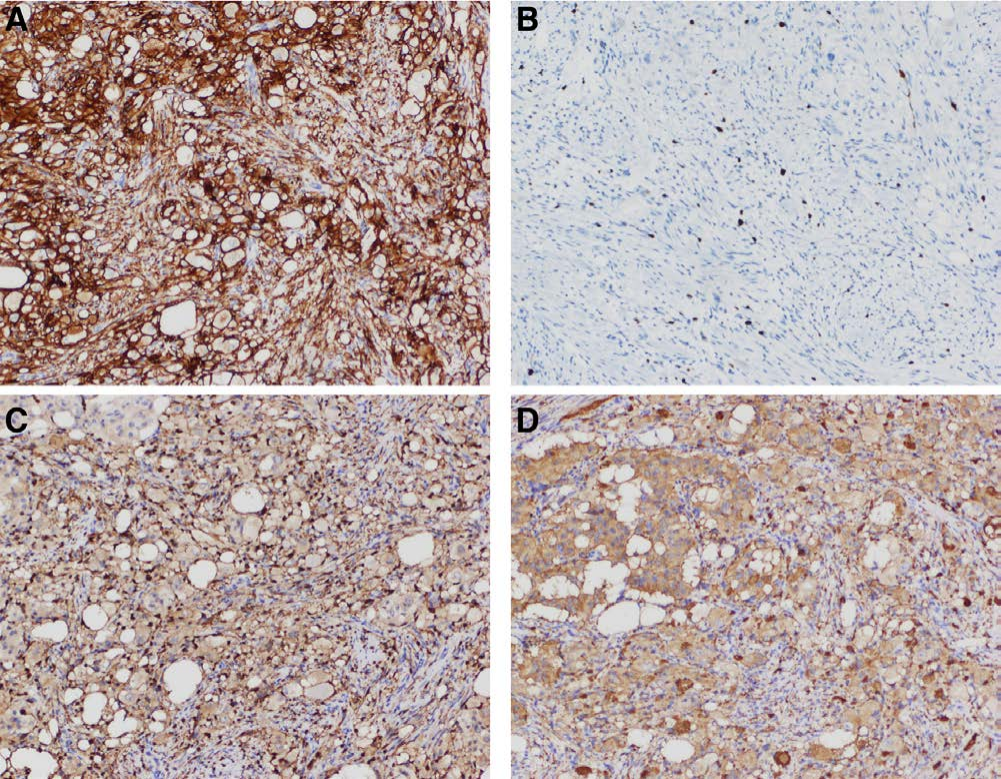
Pathological immunohistochemistry. (A) CD56(+). (B) Ki-67 (5%+). (C) S-100 (+). (D) Syn (+). Syn = synaptophysin.

A 54-year-old woman was admitted to the hospital on March 24, 2021 with the chief complaint of “black stools for 6 days.” Six days prior to admission, she had formed black stools, 1 to 2 times/day, with dizziness, fatigue, no blood vomiting, no abdominal pain, no yellow eyes, yellow urine, yellow skin, etc. The local hospital examination showed that “blood routine: white blood cell count 3.87 × 10^9/L, hemoglobin value is 47 g/L, platelet count is 249 × 10^9/L; fecal occult blood test is positive; electronic gastroscopy suggests that the duodenal main papilla tumor.” The patient had a history of gastric ulcer bleeding 10 years ago (specific diagnosis and treatment unknown); history of hypertension for more than 6 years, without regular medication and blood pressure monitoring. Physical examination on admission suggested anemic appearance and the abdomen was soft and there was no pressure or rebound pain in the whole abdomen. Adjunctive examination suggested that the blood routine: white blood cell count was 4.09 × 10^9/L, hemoglobin count was 52 g/L, INR and biochemical routine were all within the normal range; NT-BNP, cTn-T, and tumor markers (CEA, CA125, CA19-9, and AFP) were within the normal range; electronic duodenoscopy suggested that a shallow ulcer could be seen in the main papilla of the descending duodenum, about 3.0 cm*2.5 cm subpapillary round mass, about 3.0 cm*2.5 cm, with most of the mucus on the surface of the mass, most of the mucosa on the surface of the mass was in line with the surrounding intestinal wall, and a shallow ulcer was visible next to the opening of the papilla, with clear boundaries and a clean base, and no abnormal blood vessels or glandular ducts were seen in the base of the ulcer under the Flexible Spectral Imaging Color Enhancement mode of the electronic endoscopy (Fig. 1). Endoscopic biopsy pathology suggested chronic inflammation of the mucosa and low-grade intraepithelial neoplasia of the adenoepithelium; in addition, magnetic resonance imaging scanning and enhancement examination of the epigastrium suggested that a patchy and slightly higher enhancement foci were seen in the duodenal papillary area in the venous phase and the delayed phase, and magnetic resonance cholangiopancreatography suggested that there was no dilatation of the common bile ducts, the left and right hepatic ducts, or pancreatic ducts, with a low confluence of the cystic duct, and that the end of the biliopancreatic ducts did not have any obvious involvement of the mass (Fig. 2). After the patient was admitted to the hospital, he was treated with infusion of suspended red blood cells and acid production, and the biopsy of the lesion site was reexamined under the electron gastroscope 1 week later, suggesting that a small amount of fragmented glandular epithelium with intraepithelial neoplasia-like changes of undetermined significance was seen in the tissue of the duodenal papillae, which was limited in observation, and it was recommended that a larger piece of tissue be reexamined and sent to us for examination (Fig. 3). After fully communicating with the patient and his family about his condition, the patient and his family requested further endoscopic treatment, so “endoscopic papillectomy + endoscopic retrograde cholangiopancreatography + endoscopic biliary stent placement + endoscopic nasobiliary drainage” was performed on March 28, 2021. The gross specimen after surgery suggested a duodenal papillary mass measuring 3.0*2.5*1.2 cm, grayish-white in color and medium in texture, with mucosal tissue attached; microscopically, the mass was mainly located in the submucosa, with partial infiltration of the muscularis propria, and epithelioid, spindle, and ganglion cells could be seen (Fig. 4). Immunohistochemistry of the pathologic specimen showed synaptophysin (Syn) (positive), CD56 (positive), chromogranin A (a few weak positives), Ki67 (5% positive), S-100 (positive), CKpan (partially weakly positive), CK8/18 (partially weakly positive), CK5/6 (negative), and Calponin (negative) (Fig. 5). The final pathologic diagnosis was DGP. The patient was discharged after the postoperative removal of the nasobiliary drain and returned to the hospital 2 months later to have the biliary stent removed; the patient was in good general condition at follow-up.

## 3. Discussion

Gangliocytic paraganglioma is a rare neuroendocrine tumor (NET) that can occur in organs such as the lungs, mediastinum, esophagus, jejunum, pancreas, low-level spinal cord, and appendix, with a particular predilection for the duodenum. DGP was first reported by Dahl in 1957, and now the relevant data show that about 90% of them occur in the descending and horizontal part of the duodenum, and the peripelvic area is the most common one. It is well known that the jugular abdomen is the junction of the biliopancreatic duct and the duodenum, and the associated diseases are mostly acute and critical, and their management is often difficult. DGP has been regarded as a benign NET, but in recent years, cases of DGP with lymph node or distant metastasis have been reported, so it is necessary to comprehensively re-conceptualize DGP.

DGP is prevalent in adult males, and the first manifestations of DGP include gastrointestinal bleeding, anemia, abdominal pain, or biliary and intestinal obstruction, etc. Park et al^[2]^reported that the average tumor size of DGP was 2.57 cm, and the depth of tumor infiltration often exceeded the submucosal layer. Since the mucosal layer at the lesion is often not invaded by the tumor, its biopsy positivity rate is only 11.4%.^[3]^ The situation of our case in terms of tumor size, depth of infiltration, and preoperative biopsy positivity was in general agreement with related reports. Among the reports on the malignant biological behavior of DGP, Okubo^[4]^ et al showed that LNM occurred in about 11.4% and liver metastasis in 1.1%, where the main risk factors for LNM were mean tumor size and infiltration depth. Univariate analysis showed that the larger the tumor, the higher the risk of LNM for those whose infiltration depth penetrated the submucosa or sphincter of Oddi, while multifactorial logistic regression analysis indicated that the depth of infiltration of the tumor was a more important risk factor for LNM. The study also suggested that there was no significant relationship between mean tumor size and depth of tumor infiltration.

Studies on the pathogenesis of GP are still scarce. The WHO (2019)^[5]^ Classification of Tumors of the Digestive System suggests that GP may be a pancreatic progenitor-based missense tumor, which is also supported by the study of Okube et al.^[4]^ In addition, Zhuang et al^[6]^ detected somatic HIF-2α mutations in the tissues of 10 GP patients and found that 2 of them had mutations in exon 12 of the HIF-2α gene (i.e., T519 M and P544 S), and the above 2 mutation sites could lead to enhanced stability and impaired ubiquitination and degradation, which could enhance the triggering of its downstream regulatory genes and further up-regulate the HIF signaling pathway. Tatangelo et al^[7]^ suggested that NeuroD1 protein was overexpressed in GP tumors, while NeuroD1 gene expression was upregulated, suggesting that GP may be associated with abnormalities in this gene.

In terms of auxiliary examinations, relevant studies^[8]^ showed that DGP on CT showed a soft tissue parenchymal density shadow with clear boundaries, mostly protruding into the lumen, and gradual homogeneous enhancement on enhancement, while Okubo^[4]^et al found that DGP on white light endoscopy showed a bulging polypoid or nodular submucosal mass with or without mucosal erosive ulceration, and the surface mucous membrane of the patients without erosive ulceration was smooth, and some of them had a granular appearance; while Sekine^[9]^et al found that DGP often showed isoechoic and hyperechoic lesions in the submucosal layer under endoscopic ultrasonography (EUS). While Sekine et al observed that DGP was often characterized by isoechoic and hypoechoic lesions with a predominantly submucosal layer under EUS. Pathology is the gold standard for diagnosis, and Okubo^[4]^et al pointed out that DGP has 3 characteristic tumor components: epithelioid cells, ganglion cells, and spindle cells, and its immunohistochemical features are as follows: positive markers for epithelioid cells include CD56, Syn, neuron-specific enolase (NSE), progesterone receptor, pancreatic polypeptide, growth inhibitory hormone, chromogranin A, and cytokeratin. Positive markers for ganglion cells include CD56, Syn, NSE, and growth inhibitor; S-100 is the most common positive marker for spindle cells, followed by NSE, CD56, and Syn.

Despite the corresponding clinical and imaging features of DGP, misdiagnosis still exists from time to time. Clinical attention should be paid to differentiate from the following diseases: NET G1: one of the most important diseases to be differentiated from DGP, which is often manifested as a characteristic stellate soft-tissue density mass on CT, with fast-in-fast-out sign after enhancement, and heterogeneous hypoechoicity on EUS, with negative pancreatic polypeptide and progesterone receptor levels. Mesenchymal tumors: Mostly grow out of the cavity, necrosis and cystic degeneration are common, CT can show progressive enhancement, EUS is mostly border irregular hypoechoic, mostly originated from the intrinsic muscle layer, immunohistochemistry can be differentiated. Gangliocytic neuroma: benign tumors occurring in the colorectum and ileum, CT is not enhanced or mildly enhanced, pathologically lack of epithelioid cell nests. In addition to the above diseases, DGP should be distinguished from smooth muscle tumor, duodenal poorly differentiated adenocarcinoma, duodenal papillary adenoma, etc, which can be supported by endoscopic manifestations, pathology and immunohistochemistry.

There is a basic preference for both endoscopic and surgical (laparoscopic or open surgery) treatment of DGP. Barret et al^[10]^proposed the following criteria for the treatment of DGP: Diameter < 2 cm without peripheral lymph node involvement as confirmed by abdominal CT can be treated by endoscopic mucosal resection (EMR), endoscopic papillectomy (EP), or combined with translaparoscopic duodenal tumor resection with preoperative duodenoscopy; Pancreaticoduodenectomy combined with lymph node dissection should be performed if LNM is suspected or if the histopathologic features are characterized by infiltrative margins or growth beyond the submucosal layer, and if the nuclear pleomorphism or mitotic activity is high. Currently, it is generally accepted that the presence or absence of metastasis is the criterion for determining the benign or malignant nature of DGP. Considering the traumatic and risky nature of surgery near the jugular abdominal region, and the fact that many studies have supported that the risk of LNM and distant metastasis of DGP is relatively low, coupled with the development of endoscopic diagnostic and therapeutic technology, more and more scholars are employing endoscopic techniques (e.g., EMR/endoscopic submucosal dissection [ESD]/EP) to manage DGP after adequate preoperative evaluation to rule out metastatic signs and have achieved good results. Palomino-Martinez et al^[11]^ and Ebi et al^[12]^ successfully resected DGP near the nipple using EMR; Manglekar et al^[13]^ successfully resected DGP near the nipple using ESD; Sanchez-Pobre et al^[14]^ successfully managed DGP near the nipple after gastrectomy using endoscopic resection + EST; Arif et al^[15]^ and Papaconstantinou et al^[16]^ used duodenotomy to locally resect DGP near the nipple, and all of the above reports achieved satisfactory results, but some of them had postoperative complications such as cholangitis or pancreatitis. In the present case, postoperative complications were effectively prevented while the papillary DGP was managed by EP combined with endoscopic retrograde cholangiopancreatography technique. Regarding the postoperative follow-up, it is recommended that the follow-up period should be shortened for those who received endoscopic treatment (12 months on average) and extended for those who received surgical treatment (26.92 months on average), and the most common methods for review are gastroduodenoscopy and CT.

In summary, DGP is a type of NET with potentially malignant biological behavior that develops in the descending or horizontal portion of the duodenum, and the prognosis is generally good. The difference between its benign and malignant nature lies in the presence or absence of metastasis, and the high-risk factors for metastasis mainly lie in the size of the tumor and the depth of infiltration. Preoperative evaluation of the tumor and surrounding conditions by enhanced CT or EUS, and selection of appropriate endoscopic techniques (e.g., EMR/ESD/EP or even combined endoscopic retrograde cholangiopancreatography) or surgery in conjunction with the diseased portion is an optimal solution for DGP.

## Author contributions

**Resources:** Wenpin Cai.

**Writing – original draft:** Wenpin Cai, Weitao Hu, Taiyong Fang.

**Writing – review & editing:** Wenpin Cai, Weitao Hu, Taiyong Fang.
